
JOURNAL INFORMATION
==============================
NLM Title Abbreviation: Ann R Coll Surg Engl
Journal ID: Ann R Coll Surg Engl
NLM Journal ID: 7506860
Journal ID: ann

Annals of The Royal College of Surgeons of England

ISSN: 0035-8843
EISSN: 1478-7083
Publisher: Royal College of Surgeons of England

ARTICLE INFORMATION
==============================
PMCID: PMC11847618
DOI: 10.1308/rcsann.2025.0011
Article ID: rcsann.2025.0011
Article version: 1
Subjects: ASiT Innovation Summit e-Posters

ASiT Innovation Summit e-Posters

Print publication date: 2025 Feb 24
Volume: 107
Issue: Suppl 1
First page: S15
Last page: S113
Copyright: Copyright © 2025, The Authors
Copyright year: 2025
License: This is an open-access article distributed under the terms of the Creative Commons Attribution 4.0 International License, which permits unrestricted use, distribution, reproduction, and adaptation in any medium, provided the original work is properly attributed.
License URL: https://creativecommons.org/licenses/by/4.0/

==============================

Subjects: Artificial Intelligence in Surgery

83 The place for artificial intelligence in surgery

Mbashila N.
University of Lusaka, Chongwe, Zambia

141 SMART theatres: review audit of estates failures vs. SMART theatres

Hepworth Lloyd F. 1 2
Montero R. 1
Roskams D. 1
Hawchar K. 1
Coomber R. 1
1 St George's Hospital, London, United Kingdom
2 St Peter's Hospital, Chertsey, United Kingdom

143 Using machine learning to identify rapid pain progression in knee osteoarthritis

Tooke J. 1 2
Musbahi O. 1
Jones G. 1
1 Imperial College London, London, United Kingdom
2 University of Manchester, Manchester, United Kingdom

159 ChatGPT – integrity: artificial intelligence misuse in medical writing

Fay T. 1
Homewood D. 2 3
Fay D. 4
Niall O. 2 5
1 Department of Urology, Department of Surgery, Frankston Hospital, Frankston, Australia
2 International Medical Robotics Academy, Melbourne, Australia, Melbourne, Australia
3 Department of Urology, Royal Melbourne Hospital, Melbourne, Australia, Melbourne, Australia
4 Northern Hospital, Melbourne, Australia, Melbourne, Australia
5 Department of Urology, St. Vincent's Hospital Melbourne, Melbourne, Australia, Melbourne, Australia

222 Artificial intelligence – a help or hindrance in surgical training?

Bhakar R.
Torbay Hospital, Torquay, United Kingdom

229 AI-enhanced urology writing: transforming the landscape of training, scholarly progression, and academic excellence

Al-Ghazawi M.
NHS Greater Glasgow and Clyde, Glasgow, United Kingdom. Barts NHS Health, London, United Kingdom

343 Automated assessment of sarcopenia with Hounsfield unit average calculation in computed tomography scans using deep learning techniques

Rengan V.S. 1
Arora E. 2
Sundaram P.M. 3
Rengan R.S. 4
Sekaran P. 5
Kalla R. 6
Bawa A. 7
Alexander N. 8
Venkataramanan R. 9
1 SMS Medical College, Jaipur, India
2 Grant Medical College and Sir JJ Group of Hospitals, Mumbai, India
3 Apollo Specialty Hospitals, Chennai, India
4 Chennai Hernia Center, Chennai, India
5 Basildon University Hospital, Basildon, United Kingdom
6 Curium Life Tech, Chennai, India
7 Dayanand Medical College and Hospital, Ludhiana, India
8 Sri Ramachandra Institute of Higher Education and Research, Chennai, India
9 Advantage Imaging and Research Institute, Chennai, India

354 CT perfusion in a 3rd-level hospital

Alabi A.
Connolly Hospital Blanchardstown, Dublin, Ireland

404 Artificial intelligence and machine learning in sarcopenia assessment within vascular surgery: a systematic review

Maan B. 1
Tay T. 2
Nair A. 1
El-Sayed T. 3
1 Newcastle University, Newcastle upon Tyne, United Kingdom
2 Institute of Global Health Innovation, Imperial College London, London, United Kingdom
3 Newcastle Upon Tyne Hospitals, Newcastle upon Tyne, United Kingdom

415 Artificial intelligence is inferior to surgeons at assessing for the presence of reporting bias (spin) in orthopaedic sports medicine literature

Ó Mathúna E.C.E. 1
Doyle T.R. 1
Hurley E.T. 2
Klifto C.S. 2
Dickens J.F. 2
Mullett H. 1
1 UPMC Sports Surgery Clinic, Dublin, Ireland
2 Duke University Medical Centre, Durham, USA

Subjects: Clinical Research

3 The evaluation of blood parameters for the reduction of negative appendectomy

Bin Ihsan S. 1
Shakeel O. 2
Rajpal P. 2 3
1 Trinity College Dublin, Dublin, Ireland
2 Cavan General Hospital, Cavan, Ireland
3 Royal College of Surgeons Ireland, Dublin, Ireland

34 Assessing postoperative pain in total knee arthroplasty patients using an automated self-logging patient-reported outcome measure collection device: a retrospective cohort study

Ajrawat P. 1
Price B. 2
Gooch D. 2
Serban R. 3
Al-Habsi R. 4
Pearce O. 3
1 University of Buckingham Medical School, Buckingham, United Kingdom
2 School of Computing & Communications, The Open University, Milton Keynes, United Kingdom
3 Trauma and Orthopaedics Department, Milton Keynes University Hospital, Milton Keynes, United Kingdom
4 Trauma and Orthopaedics Department, Barts Health NHS Trust, London, United Kingdom

35 Publication trends in aesthetic breast surgery - a bibliometric analysis

Rupra R. 1
Daneshi K. 2
Liyanage D. 3
Ceccaroni A. 4
Gentile A. 4
Khajuria A. 5 6
1 Department of Surgery, James Paget University Hospitals NHS Foundation Trust, Great Yarmouth, United Kingdom
2 School of Medicine and Population Health, The University of Sheffield, Sheffield, United Kingdom
3 Department of Surgery, Queen Elizabeth Hospital, Birmingham, United Kingdom
4 Plastic Surgery Unit, Department of Medicine, Surgery and Dentistry, University of Salerno, Salerno, Italy
5 Department of Surgery & Cancer, Imperial College London, London, United Kingdom
6 Kellogg College, University of Oxford, Oxford, United Kingdom

53 Comparative study of sternal closure devices post adult cardiac surgery

Keating T. 1
Tripathy A. 2
Larobina M. 2
Skillington P. 2
1 St James Hospital, Dublin, Ireland
2 Royal Melbourne Hospital, Melbourne, Australia

59 Twisted gut, stricken liver: a case of ischemic hepatitis from caecal volvulus

Shaikh A.G. 1
Saunders J. 2
1 Kingston Hospital, London, United Kingdom
2 Royal Victoria Infirmary, Newcastle, United Kingdom

78 Predictors of intraoperative blood transfusion in elective surgeries at different hospitals of Addis Ababa. A multi-centre cross-sectional analytical study

Denberu Y. 1
Melaku R.T. 2
1 Addis Abeba, Addis Abeba, Ethiopia
2 Addis Ababa University, Addis Abeba, Ethiopia

96 Comparative study of ALVARADO score and RIPASA score in the diagnosis of acute appendicitis

Ebrahim Mohamed ElMoslimany A.
Al_Azhar University, Cairo, Egypt

103 Comparative study between fissurectomy and lateral sphincterotomy versus fissurectomy and anal dilatation in management of chronic

Ebrahim Mohamed ElMoslimany A.
Al_Azhar university, Cairo, Egypt

109 Identifying unmet needs concerning luminal device insertion in surgery – a pan-speciality cross-sectional survey and qualitative analysis

Aloul Z. 1
Mills E. 2
Burke J. 3
1 South Tees Hospitals Foundation Trust, Middlesborough, United Kingdom
2 Mid and South Essex NHS Foundation Trust, Essex, United Kingdom
3 Manchester Royal Infirmary, Manchester, United Kingdom

111 Impact of bariatric surgery on obesity indices and reproductive hormones: A prospective study in morbidly obese women

Almoshantaf M.B. 1
Bananis K. 2
Voros C. 3
1 Ealing Hospital, LNWH Trust, London, United Kingdom
2 King's College, London, United Kingdom
3 National and Kapodistrean University of Athens, Athens, Greece

146 Evaluation of the impact of an enhanced recovery pathway on clinical outcomes after pancreaticoduodenectomy

Ugur S.
Armstrong T.
Clarke H.
Fraser J.
Hamady Z.
Karavias D.
Pike T.
Takhar A.
Arshad A.
Southampton General Hospital, Southampton, United Kingdom

148 Assessing outcomes and complications of laparoscopic cholecystectomy: A retrospective study

Khalid S.Y. 1 2
Elamin A. 2
O’Shea D. 2
Bodnar Z. 2
1 St. James's Hospital, Dublin, Ireland
2 Letterkenny University Hospital, Letterkenny, Ireland

164 Can neutrophil-lymphocyte ratio be a reliable early predictor of the severity of acute pancreatitis?

Anwar S.
Rashid Hospital, Dubai Health Authority, Dubai, UAE

198 Is pain and mental health correlated in patients with scoliosis completing physiotherapy scoliosis specific exercise?

Gower D.
Pilcher C.
St. George's University Hospital NHS Trust, London, United Kingdom

207 Evaluating the impact of PSA density on clinically significant prostate cancer detection in PIRADS-3 lesions – a single-centre study

Safdar H.
Medway NHS Foundation Trust, Gillingham, Kent, United Kingdom

208 Prostatic adenocarcinoma in a patient with history of cryptorchidism associated hypogonadism - a case report

Safdar H.
Medway NHS Foundation Trust, Gillingham, Kent, United Kingdom

213 Outcomes of common peroneal nerve neurolysis following total knee arthroplasty-related injury

Stewart N. 1
Ali Y. 2
Fox M. 1
McCulloch R. 1
1 Royal National Orthopaedic Hospital, London, United Kingdom
2 University College London, London, United Kingdom

235 Ten-year experience with cecal volvulus – a retrospective single-center study

Sekaran P.
Bashir N.
Kumar S.
Ashraf N.
Basildon University Hospital, Mid and South Essex NHS Foundation Trust, Basildon, United Kingdom

240 Fournier’s gangrene caused by candida albicans: A case report

Amusat O.A.
Bodean V.
Mohammed A.
Luton and Dunstable University Hospital, Luton, United Kingdom

243 Maximising recovery: evaluating the impact of incentive spirometers on post-cardiac surgery outcomes

Kallikere Lakshmana S.K.
Vlachou C.
Tamang R.
Hammamieh A.
Trimlett R.
Yusuf M.
Elkholy A.
Royal Brompton Hospital, London, United Kingdom

254 Evaluating the diagnostic accuracy of MRI-derived PSA density in prostate cancer detection and its association with tumour aggressiveness

Khalid S.Y. 1 2
Waraich T.A. 2
Elamin A. 2
1 St. James's Hospital, Dublin, Ireland
2 Letterkenny University Hospital, Letterkenny, Ireland

266 Neck anatomy explored with 993 CT scans

Lynch C. 1
Abrar R. 2
1 Stepping Hill Hospital, Stockport, United Kingdom
2 Wythenshawe Hospital, Manchester, United Kingdom

280 Predicting the post-surgical resumption of physical activities in adolescent idiopathic scoliosis

Elhariry M. 1
Khaleeq T. 1
Jaly I. 2
Lea M. 3
Tokala P. 3
Davidson N. 3
Trivedi J. 3
Munigangaiah S. 3
1 Sandwell General Hospital, Birmingham, United Kingdom
2 Royal Orthopaedic Hospital, Birmingham, United Kingdom
3 Alder Hey Children's Hospital, Liverpool, United Kingdom

281 Hemi end-to-end and end-to-end techniques for nerve transfers in high ulnar nerve transection

Weekes M.
Burahee A.
George S.
Beale S.
McGhee C.
Power D.M.
University Hospitals Birmingham NHS Foundation Trust, Birmingham, United Kingdom

286 Evaluating outcomes for branch of medial head of triceps nerve transfer to axillary nerve to improve abduction and external rotation at the shoulder

Weekes M.
Jimulia D.
Malone P.
McGhee C.
Power D.M.
University Hospitals Birmingham NHS Foundation Trust, Birmingham, United Kingdom

315 Renal calculi in proteus urinary tract infection – is the search for stones always required?

Tyler G.
Durbidge C.
Patel K.
Rudd I.
Maidstone and Tunbridge Wells NHS Trust, Maidstone, United Kingdom

324 Use of smartphone activity data to monitor and identify trends in recovery after breast reduction surgery

Tupper L.
Kwasnicki R.
Wood S.
Imperial College NHS Trust, London, United Kingdom

341 An uncommon case of spontaneous tension haemopneumothorax in a young man with initially stable observations

Fowler C.
Qsous G.
McSorely M.
Elbakbak W.
Will M.
Royal Infirmary Edinburgh, Edinburgh, United Kingdom

368 Volume restoration and mastopexy following breast implant removal using auto-augmentation with dual pedicles: a case series

Abdelaty M. 1
Ashfaq F. 2
1 Bradford teaching hospitals NHS Foundation trust, Bradford, United Kingdom
2 University Hospital of North Midlands, Stoke, United Kingdom

369 Gender-specific associations between extremity adiposity and depression risk in individuals without central obesity

Wong Z.Y. 1
Ou K.Q. 2
Faderani R. 3
Kanapathy M. 3
Mosahebi A. 3
1 Morriston Hospital, Swansea, United Kingdom
2 Southmead Hospital, Bristol, United Kingdom
3 UCL, London, United Kingdom

373 Spontaneous calyceal rupture due to a 3mm obstructing ureteric stone: a case report

Waraich T.A. 1
Khalid S.Y. 2 1
Muhammad O. 1
Omer S. 1
1 Letterkenny University Hospital, Letterkenny, Ireland
2 St. James's Hospital, Dublin, Ireland

378 A retrospective cohort study assessing ability of radiological investigations to predict axillary nodal status in breast cancer

Bashir Ni.
Mid and south Essex NHS trust, Basildon, United Kingdom

396 Incisionless otoplasty case series: a safe and effective minimally invasive technique for prominent ear correction

Abdelaty M. 1
Ashfaq F. 2
1 Bradford Teaching Hospitals NHS Foundation Trust, Bradford, United Kingdom
2 University Hospitals of North Midlands, Stoke, United Kingdom

398 Epidemiological characteristics, incidence trends, and survival outcomes of nasopharyngeal carcinoma (2000–2021): a comprehensive analysis of 10,419 cases

Khalil A.M.A. 1
Allam A.R. 2
Helal M.B. 3
Algohary R. 3
Akbar S. 4
Elsayed M.N. 5
1 Royal Derby Hospital, Derby, United Kingdom
2 Faculty of Medicine, Menoufia University, Menoufia, Egypt
3 Faculty of Medicine, Menoufia University, Menoufia, Egypt
4 North West Deanery, Manchester, United Kingdom
5 Stockport NHS Foundation Trust, Stockport, United Kingdom

400 Is there an association between intracranial pressure (ICP) and patient-reported symptoms in adult patients with CSF disorders

Tagdiwala P. 1
Dhaliwal J. 1
Pandit A. 2
Vicedo C.K. 2
Thompson S. 2
Thorne L. 2
Watkins L. 2
Toma A. 2
1 University College London Medical School, London, United Kingdom
2 National Hospital for Neurology and Neurosurgery, London, United Kingdom

416 Neurosurgery journals and utilisation of social media: a bibliometric analysis

Burton O. 1 2
Asif A. 3
Panchal H. 4
Basra R. 2
1 Healthcare Quality Improvement Partnership (HQIP), London, United Kingdom
2 University College London Hospitals NHS Trust, London, United Kingdom
3 Division of Surgery and Interventional Science, University College London, London, United Kingdom
4 University College London (UCL), London, United Kingdom

417 Appendiceal mucocele - a review of small cohort

Kumar S.
Alkistawi F.
Basildon University Hospital, Basildon, United Kingdom

418 Gallstone pancreatitis : timing of surgery is critical

Unadike C. 1
Namme E. 2
1 Isle of Wight, Isle of Wight, United Kingdom
2 University Hospital Southampton, Southampton, United Kingdom

450 Complete response of metastatic right colon cancer after pembrolizumab treatment and emergency right hemicolectomy with anastomosis: a case report

Gajre M.
Rahim A.
Maidstone and Tunbridge Wells NHS Trust, Kent, United Kingdom

457 Are online resources trustworthy and do the public understand them? Assessing the quality and readability of online material for facial fat transfer

Mackay J. 1
Dadabhoy M. 2
1 Nottingham University Hospitals NHS Trust, Nottingham, United Kingdom
2 Royal Free Hospital NHS Trust, London, United Kingdom

462 Trampoline associated injuries in paediatric patients: patterns, management, and implications

Bakko F.
Maxwell M.
Zakaria B.
Taylor L.
Sawyer L.
Sharma R.
Epsom and St Helier University Hospitals NHS Trust, Carshalton, United Kingdom

466 Effect of operation type and pre-operative demographic factors on weight-loss and weight regain after bariatric surgery: a longitudinal study from a public healthcare perspective

Bin Aizan L.N. 1
Alkhaffaf B. 1 2
1 Department of Oesophago-Gastric and Bariatric Surgery, Salford Care Organisation, Northern Care Alliance NHS Foundation Trust, Greater Manchester, United Kingdom
2 Faculty of Biology, Medicine and Health, University of Manchester, Manchester, United Kingdom

470 Magnetic resonance imaging findings in suspected cauda equina syndrome referrals: epidemiological insights into diagnostic patterns and clinical correlations

Mceneaney L.
Maxwell M.
Zhang J.
Maheswaran A.
Khan I.
ElNawasany M.
Abdel-Hafez Y.
Day A.M.
Hulme C.
Epsom and St Helier University Hospitals NHS Trust, Carshalton, United Kingdom

474 Does anti-factor Xa monitoring reduce surgical timing in neck of femur patients on direct oral anticoagulant therapy

Maxwell M. 1
Horlick S. 2
Hau M. 3
Nichols J. 3
1 Epsom and St Helier University Hospitals NHS Trust, Carshalton, United Kingdom
2 Kingston Hospital NHS Foundation Trust, Kingston, United Kingdom
3 University Hospitals of Leicester NHS Trust, Leicester, United Kingdom

482 Direct comparison of pneumatic and laser lithotripsy in the management of lower ureteric stone-in Ibn Sina hospital and Omdurman military hospital

Mohammed E. 1
Greeballah A. 2
1 Red Sea University Faculty of Medicine, Portsudan, Sudan
2 Sudanese Medical SIpecialization Board SMSB, Khartoum, Sudan

483 Predictors of 30-day mortality following emergency laparotomy

Hassan M.
Abdelsaid K.
Jayasankar B.
Maidstone and Tunbridge Wells NHS trust, Tunbridge Wells, United Kingdom

490 To biopsy or not to biopsy – diagnostic value of post nasal space biopsy for vasculitis

Jaffer M.
Diver C.
Royal Victoria Hospital, Belfast, United Kingdom

493 Appraisal of current surgical guidelines for inflammatory bowel disease (IBD) using the AGREE-S instrument: A scoping review

Khan Z. 1
Ball C. 2
Saeed D. 2
Tai G. 2
Chandran S. 2
Vashista A. 2
Davey S. 1
Lee M. 3
Brown S. 1
Hind D. 2
Sayers A. 1
1 Sheffield Teaching Hospitals, Sheffield, United Kingdom
2 University of Sheffield, Sheffield, United Kingdom
3 University of Birmingham, Birmingham, United Kingdom

Subjects: Collaborative Research

212 Global outcomes in athletes after cardiac event ‘’GOACE’’ protocol: an international retrospective collaborative study assessing the global variation in patient characteristics, management, and outcomes of elite athletes following cardiac events and heart surgery.

Eldoadoa M. 1 2
Hamraa J. 3 2
Tran T.Q.M. 4 2
Balouz M.A. 5 2
Tabash S.A. 6 2
Elbashir W.E.E. 7 2
Huy N.T. 8 2
1 St Helens & Knowsley NHS Teaching Hospital Trust, Merseyside, Japan
2 Online Research Club, Nagasaki, Japan
3 International Islamic university, Islamabad, Pakistan
4 Washington University School of Medicine in St. Louis, MO, Missouri, USA
5 Faculty of Medicine Menoufia university, Menoufia, Egypt
6 Ain Shams University Faculty of Medicine, Cairo, Egypt
7 Alzaiem Alazhari university Faculty of Medicine, Khartoum, Sudan
8 School of Tropical Medicine and Global Health (TMGH), Nagasaki University, Nagasaki, Japan

238 A comparison of the reported complications following primary joint arthroplasties by the Scottish Arthroplasty Project (SAP) and the Golden Jubilee National Hospital (GJNH) from 2014–2017

Chen G.A.
Golden Jubilee National Hospital, Glasgow, United Kingdom

385 Surveillance strategy for lung cancer and thymoma: chest X-ray or CT scan?

Guirguis P. 1
Alqudah O. 2
Vianna T. 3
Osman A. 4
Qsous G. 3
1 Liverpool Heart and Chest Hospital, Liverpool, United Kingdom
2 Norfolk and Norwich University Hospital, Norwich, United Kingdom
3 Royal Infirmary of Edinburgh, Edinburgh, United Kingdom
4 Nottingham University Hospital, Nottingham, United Kingdom

421 Insights of small bites technique in emergency laparotomy fascial closure

Inteti K.
Magee C.
Wilson J.
Wirral University Hospitals NHS Foundation Trust, Merseyside, United Kingdom

438 Feasibility study and pilot of a near-peer-led critical skills workshop for junior doctors

Curtin G. 1 2
Blake A. 1
Spring A. 1
1 University Hospital Limerick, Limerick, Ireland
2 London School of Hygiene & Tropical Medicine, London, United Kingdom

Subjects: Early-Stage Innovation

46 Revolutionising surgery: how digital prehabilitation can transform surgical patient outcomes in the NHS

Ahmed M.
Queen's Hospital, Romford, United Kingdom

112 The development of the mid-Yorkshire skin surgery simulator: low-cost and accessible simulation for the planning and raising of facial flaps

Mills K.
Magness C.
Lymperopoulos N.
Majumder S.
Plastic surgery Pinderfields Hospital, Wakefield, United Kingdom

190 Bony hand injury pathway for virtual fracture clinic - an innovative clinical management protocol

Selvarajan D.P.
Mallina R.
Spanoudakis E.
Croydon Health Services, London, United Kingdom

234 Enhancing surgical education with custom GPTs; configuring model behaviour for an ENT learning resource

Caglayan A. 1
Mehta K. 2
Balakrishnan A. 3
1 Northwick Park Hospital, London, United Kingdom
2 Ysbyty Gwynedd Hospital, Bangor, United Kingdom
3 Queen Elizabeth Hospital Birmingham, Birmingham, United Kingdom

245 Use of a novel interactive video tool in assessing UK resident doctors’ knowledge of nasendoscopic anatomy

Osuji J.
Rana M.
Maweni R.
Oxford University Hospitals, Oxford, United Kingdom

258 Gamification of CPR training for out-of-hospital cardiac arrest – a design-led approach

Curtin G.
London School of Hygiene & Tropical Medicine, London, United Kingdom. University of London, London, United Kingdom. The Digital Hub Development Agency, Dublin, Ireland

259 Implementing an electronic health record system in a resource-limited surgical oncology setting: a mixed-methods evaluation

Nellihela A.P. 1
Bandaranayake V. 1
Senevirathne R. 1
Pathirana T. 1
Gallala M. 1
Nishantha R. 1
Asanthi J. 1
Gunaratne K. 2
Senarathna M. 3
1 Teaching Hospital, Anuradhapura, Sri Lanka
2 Base Hospital, Dambulla, Sri Lanka
3 Postgraduate Institute of Science, University of Peradeniya, Colombo, Sri Lanka

260 A study of speech surgery procedures at Mater Dei Hospital, Malta

Bugeja K. 1
Parnis J. 2
Borg D. 1
1 Mater Dei Hospital, Msida, Malta
2 Mater dei Hospital, Msida, Malta

269 Using virtual simulation gamification to enhance lecturer-driven surgical education and student engagement powered by unreal engine for UKMLA spiral curriculum dissemination: a scoping review protocol

Wellington J.
Ghumman K.
Babayemi O.
Gillon C.H.M.
Sivayoganathan D.
Mentorverse, Colchester, United Kingdom

271 Silicone mould for management of auricular seroma and haematoma: a case series

Mortaja S.
Nagalotimath U.
Galli F.
Countess of Chester Hospital, Chester, United Kingdom

299 3D printing in mastoid surgery for preoperative planning

Gurney M.
Swansea Bay University Health Board, Swansea, United Kingdom

309 Syncmed: A digital platform revolutionising medical education through virtual doctor-student collaboration

Sheik-Ali A.
St George’s, London, United Kingdom

390 The impact of a student-led low-cost microsurgery conference on medical students' confidence in microsurgical techniques and career interest in plastic surgery

Singh R. 1
Mahmood A. 1
Gokani V. 2
1 School of Medicine, Imperial College London, London, United Kingdom
2 Department of Plastic and Reconstructive Surgery, Imperial College Healthcare NHS Trust, London, United Kingdom

403 Evaluating a national teaching series and understanding the ideal resources to study the UKMLA

Tagdiwala P. 1
Petmeza C.A. 2
Dubey S. 3
Murray I. 4
Arora A. 5
Kerdegari N. 5
1 University college London medical school, London, United Kingdom
2 Queens Mary University of London, London, United Kingdom
3 The University of Nottingham, London, United Kingdom
4 Queen’s University Belfast, London, United Kingdom
5 Kings College London, London, United Kingdom

412 An interaction design approach to improving patient experience of the lower limb trauma pathway

Cooper K. 1
Murphy H. 2
Quinlan C. 1
1 The Mater Misericordiae University Hospital, Dublin, Ireland
2 National College of Art and Design, Dublin, Ireland

428 Topical liposomal amphotericin B (LamB) in major burn injury with fusariosis: an innovative treatment approach

Clesham M.
Perusseau-Lambert A.
Frew Q.
St Andrew's Centre for Plastic Surgery and Burns, Chelmsford, United Kingdom

Subjects: Global surgery (at least one author from a low- and middle-income country (LMIC)

44 Indications and levels of Limb Amputation in Abakaliki, South -East Nigeria; a 5-year retrospective review

Ugochukwu I. 1
Aroh V. 2
Okoye J. 3
Uke K. 4
1 Leeds Teaching Hospitals NHS Trust, Leeds, United Kingdom
2 Alex Ekwueme University Teaching Hospital, Abakaliki, Ebonyi State, Nigeria
3 Mersey and West Lancashire Teaching Hospital NHS Trust, Prescot, United Kingdom
4 Nnamdi Azikiwe University Teaching Hospital, Nnewi, Anambra State, Nigeria

66 CT tractography as a last option for penetrating abdominal wall injuries with equivocal contrast CT findings

Ewedah M. 1
Bassiony M. 2
Asal M. 2
1 Barking Havering Redbridge University Trust, London, United Kingdom
2 Alexandria Main University Hospital, Alexandria, Egypt

76 Congenital peritoneal membrane encapsulation causing acute intestinal obstruction

Inedu P.
Oluwatosinn A.
Orisakwe C.
Yau G.
Cephas B.
National Hospital Abuja, Abuja, Nigeria

79 Addressing the burden of war-related injuries in Sana’a, Yemen: A general practitioner’s perspective

Albasi K.
Sana'a University, Sana'a, Yemen

82 Metabolic and bariatric surgery training and practice in Pakistan: identifying barriers and proposing solutions

Khan H.J. 1
Ghumman A.K. 2
Umar R. 3
Yunus T. 2
1 King Edward Medical University, Lahore, Pakistan
2 Evercare Hospital, Lahore, Pakistan
3 Jinnah Hospital, Lahore, Pakistan

108 Stomach cancer elective surgery morbidity and mortality at 90-day (HOLD study): A prospective, international collaborative cohort study

Abuobaida E.E. 1
Neves C. 2
1 National Ribat University, Khartoum, Sudan
2 University of Portugal, Lisbon, Portugal

110 To-shave or not-to-shave: impact on postoperative outcomes in haemorrhoidectomy patients; a retrospective case-controlled eminence-based study

Almoshantaf M.B. 1
Khaled Mohammed M. 2
Hamdy N.M. 3
Hussien A.F. 4
Abdelsamad A. 5
Nasif N. 6
1 Ealing Hospital, LNWH Trust, London, United Kingdom
2 General Surgery Department, Faculty of Medicine, Cairo University, Al-Kasr Al Aini, Cairo, Egypt, Cairo, Egypt
3 Biochemistry Department, Faculty of Pharmacy, Ain Shams University, Abassia, Cairo, Egypt, Cairo, Egypt
4 General Surgery Department, Mansoura University Hospitals, Mansoura, Egypt, Mansoura, Egypt
5 Section Head Robotic Surgery, Senior Consultant Robotic, Colorectal, and Oncological Surgery - Knappschaft Vest-Hospital, 45657 Recklinghausen, Germany, Recklinghausen, Germany
6 Aleppo University, Aleppo, Syrian Arab Republic

114 A peaky girl with a gigantic spleen – a case report

Halid A.
Kamaruzaman N.A.
Razali A.
Abdul Manan M.A.
Ismail N.
Thomas M.
Hospital Sultan Ismail, Johor Bahru, Malaysia

119 Minimally invasive procedure for haemorrhoids - our experience: a case series with review of literature

Dar K.H. 1
Harvitkar R. 1
Rohatgi Y. 2
Joshi A. 2
1 Queen Alexandra Hospital, Portsmouth, United Kingdom
2 Dr L H Hiranandani Hospital, Mumbai, India

126 Comprehensive literature review of primary seminal vesicle malignancy

Al-Gburi S.
Namuq Z.
Mosul University, Mosul, Iraq

129 Assessment of functional recovery after joint replacement surgery

Elobaid S. 1
Elawad M. 2
Hamrawi Nahla 3
Malik Abrar 2
Ahmed Adil 4
Aldeen Mohamed Emad 5
Amin Sami 6
Hamed Mazin 7
1 NHS, Macclesfield, United Kingdom
2 Almalik Academy of Medical Research, Khartoum, Sudan
3 Sudan Medical Specializations Board (SMSB), Khartoum, Sudan
4 CATRION Catering Holding Company, Riyadh, Saudi Arabia
5 Prince Sultan Military Medical City, Riyadh, Saudi Arabia
6 Al - Iman General Hospital, Riyadh, Saudi Arabia
7 KSAU - HS, Riyadh, Saudi Arabia

138 Traumatic tongue laceration in children: a case report and literature review

Nadhira F. 1
Amelinda D.A.A. 1 2
1 Gatot Soebroto Army Hospital, Jakarta, Indonesia
2 Chasan Boesorie Public Hospital, Maluku, Moluccas, Indonesia

140 Current updates in rhinoplasty: versatility in cartilage graft

Nadhira F.
Amelinda D.A.A.
Gatot Soebroto Army Hospital, Jakarta, Indonesia

199 A comprehensive review of recent trends in posterior malleolus fracture management

Datta S. 1
Bandyopadhyay B. 2
Bose G. 3
Mukherjee S. 4
1 East Kent University Hospitals NHS Foundation Trust, Margate, United Kingdom
2 Aneurin Bevan University Health Board, Newport, United Kingdom
3 North West Anglia NHS Foundation Trust, Peterborough, United Kingdom
4 Calcutta National Medical College and Hospital, Kolkata, India

225 Supermicrosurgery as a palliative care for lymphedema: pearls and pitfalls

Amelinda D.A.A.
Irwansyah D.
Budiman
Nadhira F.
Gatot Soebroto Central Army Hospital, Jakarta, Indonesia

231 NELA: A vital initiative in the realm of global surgery

Unadike C. 1
Itoko T. 1
Aweke G. 1
Oseghale E. 2
Rutter P. 1
1 Isle of Wight NHS Trust, isle of wight, United Kingdom
2 National Orthopaedics Hospital, Lagos, Nigeria

233 Penile fracture in Africa: A scoping review of the mechanism of injury and management

Sanni Q. 1
Solagbade R. 2
Amusat O. 3
1 South Warwickshire University NHS Foundation Trust, Warwick, United Kingdom
2 University of Ilorin Teaching Hospital, Ilorin, Nigeria
3 Luton and Dunstable University Hospital, Luton, United Kingdom

253 Facial subcutaneous reconstructive surgery after massive facial haemangioma removal: pearls and pitfalls

Harsono A.D.
Amelinda D.A.A
Gatot Soebroto Central Army Hospital, Jakarta, Indonesia

255 Uncommon presentation of calcaneal tuberculosis: a case report

Awad A. 1
Abdelrahman M. 2
Khalid M. 3
Mohammed A. 4
Abdelrahman A. 5
1 Ahfad University for Women, Khartoum, Sudan
2 Nile university, Kharoum, Sudan
3 University of Bahri, Khartoum, Sudan
4 University of Khartoum, Khartoum, Sudan
5 University of Kharto, Khartoum, Sudan

344 Unlocking AI’s potential in a low-income healthcare system: doctors’ insight on benefits and barriers

Aftab S.B. 1
Ejaz H. 2
Noor R. 1
Shahzad T. 1
1 Pakistan Institute of Medical Sciences, Islamabad, Pakistan
2 Broomfield Hospital, Chelmsford, United Kingdom

355 Diffuse mesenteric and bowel angiodysplasia

Almansour B. 1
Abbad S. 2
Albashari M. 2
Alhelal M. 3
1 King Saud Medical City, Riyadh, Saudi Arabia
2 Alfaisal University, Riyadh, Saudi Arabia
3 Alrazi University, Khartoum, Saudi Arabia

422 An audit on the adequacy of pain management in a single surgical unit: are we up to the mark?

Mubarack O.M.O. 1 2
Wickramasinghe D. 1
Wickramasinghe D. 1
1 National Hospital of Sri Lanka, Colombo, Colombo, Sri Lanka
2 Colchester General Hospital, Colchester, United Kingdom

453 Global surgery awareness and education gaps among Libyan medical students

Elmaramyi M. 1
Nagib T. 2
Altarhoni A.A.M. 2
Bin Ziqlam M. 2
Hussien M. 3
Meelad A. 4
Rawag W. 2
1 Bronglais General Hospital, Hywel dda University Health Board, Aberystwyth, United Kingdom
2 Faculty of Medicine, University of Tripoli, Tripoli, Libya
3 Department of Otolaryngology, Division of Surgery, Mälarsjukhuset Hospital, Eskilstuna, Sweden, Eskilstuna, Sweden
4 Faculty of Medicine, University of Tubruk, Tubruk, Libya

Subjects: QI and audit

4 Paediatric burns admissions at Mater Dei Hospital: a comprehensive audit

Nasser Z.
Bugeja K.
Borg D.
Grima T.
Mater Dei Hospital, Msida, Malta

5 Documented adherence to catheter standards and guidelines: an audit

Maha Q.A. 1 2
Rasheed Y. 3
Javed M. 3
Aziz W. 2
1 Nottingham University Hospital, Nottingham, United Kingdom
2 Aga Khan University Hospital, Karachi, Pakistan
3 Aga Khan University Hospital, Karachi, United Kingdom

9 What does ERAS mean to foundation doctors?

Pereira F.
Kynaston L.
Chaudhary O.
Torrance A.
Broomfield Hospital, Chelmsford, United Kingdom

10 Do or do not attempt to resuscitate?

Pereira F.
Gardner L.
Torrance A.
Chaudhary O.
Broomfield Hospital, Chelmsford, United Kingdom

13 A closed loop clinical audit assessing the accuracy of documenting risks when consenting patients for laparoscopic +/- open appendicectomy

Hamal P. 1
Raven-Gregg T. 2
Jones H. 1
Naseer F. 1
O'Reilly D. 1
1 University Hospital of Wales, Cardiff, United Kingdom
2 Morriston Hospital, Swansea, United Kingdom

14 Nose no bounds: improving nasal trauma management in emergency ENT clinic

Jovanovic I.
Antrim Area Hospital, Antrim, United Kingdom

16 Enhancing documentation practices for breast reconstruction referrals

Usurua U.
Bugeja K.
Mbaekwe E.
Grima T.
De Gabriele S.
Mater Dei Hospital, Msida, Malta

17 Acute complicated diverticulitis - a snapshot of 6-monthly data of Warwick Hospital

Bakr A.A.A.
Warwick Hospital, Warwick, United Kingdom

21 Delving into LATP biopsy infection rates: what it means for antibiotic prophylaxis

Gkaliamoutsas S.
Pumfrey N.
Wong S.
Manchester Royal Infirmary, Manchester, United Kingdom

23 Are GPs getting it right? Evaluating the accuracy of referrals to a surgical assessment unit

Mamareli C.
Chung L.
General Surgery Department, Inverclyde Royal Hospital, NHS Greater Glasgow and Clyde, Greenock, United Kingdom

24 Penetrating neck trauma and rising rates of deliberate self-harm: a single trauma centre experience

Qasem M.
Ryan M.
Davies T.
Kinshuck A.
Liverpool University Hospitals Foundation Trust, Liverpool, United Kingdom

29 Perioperative risk assessment in emergency general surgery: a single centre two-cycle audit

Georgi M. 1 2
Marles H. 1
Abdullah S. 1
Smith J. 1
1 West Middlesex University Hospital, London, United Kingdom
2 Imperial College London, London, United Kingdom

33 Analysis of trends of antibiotic use in the treatment of acute diarrhoea in the emergency department: a quality improvement initiative

Fayyaz H. 1
Faheem M. 2
Khawaja M.F. 3
1 Colchester General Hospital, Colchester, United Kingdom
2 Royal Cornwall Hospital, Truro, United Kingdom
3 University Hospital Coventry & Warwickshire, Coventry, United Kingdom

37 Evaluating the differences in costs and complication rates between suture ligation and surgical excision in treating paediatric ulnar polydactyly

Robinson A. 1
Terence K. 1
Kang N. 2
Zargaran D. 2
Kokkinos C. 2
1 University College London, London, United Kingdom
2 Royal Free Hospital, London, United Kingdom

38 Role of true axial x-ray in management of ACJ injuries – QIP

Basha A.
Varashi T.
Baugley M.
Northampton General Hospital, Northampton, United Kingdom

40 Enhancing patient awareness and preparedness for flexible cystoscopy: a quality improvement project

Hunt A.
Vanalia P.
Mohee A.
Manchester Royal Infirmary, Manchester, United Kingdom

42 Can a patient's first contact with the clinic impact their overall satisfaction with the visit?

Elghouneimy M.
Khalaf A.
Manchester University NHS Foundation Trust, Manchester, United Kingdom

43 Treatment escalation plan (TEP) completion rates in emergency surgical admissions: a closed loop audit

Riad A.
Atkinson I.
Nicolay C.
West Middlesex University Hospital, Chelsea & Westminster NHS Foundation Trust, London, United Kingdom

45 Can we better streamline inpatient referrals to two-week wait colorectal cancer clinics?

Armstrong D.
Yamada K.
Abounozha S.
Coates R.
Sunderland Royal Hospital, Sunderland, United Kingdom

47 Early diagnosis and pre-admission care of fractured neck of femur

Dutta S.
Dey O.
Frimley Park Hospital, Camberley, United Kingdom

51 Assessing adherence to Royal College of Surgeons guidelines: a closed-loop audit of operation notes

Ang K.L. 1
Hosten K. 1
Behera K. 2
Chen S. 1
Tenovici G. 3
1 Oxford University Hospital, Oxford, United Kingdom
2 Bart’s Health and Queen Mary University London, London, United Kingdom
3 Milton Keynes University Hospital, Milton Keynes, United Kingdom

52 Quality of documentation of urinary catheterisation in surgical wards

Khattak M.A.
Shah M.
Dukic I.
Heartlands Hospital, University Hospitals Birmingham NHS Foundation Trust, Birmingham, United Kingdom

56 Compliance with BURST-BAUS consensus document for scrotal explorations for suspected testicular torsion: a single centre audit and QIP

Safdar H.
Medway NHS Foundation Trust, Gillingham, Kent, United Kingdom

57 A closed loop AUDIT analysing the efficacy of HEPMA electronic prescribing for medication reconciliation in a single centre colorectal surgery ward

May 1,2,3 V.
Ramsay 1,2,3 G.
1 University of Aberdeen Medical School, Aberdeen, United Kingdom
2 Aberdeen Royal Infirmary, Aberdeen, United Kingdom
3 Health Services Research Unit, Aberdeen, United Kingdom

58 Revolutionizing consent for laparoscopic cholecystectomy: breakthroughs from a two-cycle audit

Shaikh A.G. 1
Saunders J. 2
1 Kingston Hospital, London, United Kingdom
2 Royal Victoria Infirmary, Newcastle, United Kingdom

64 Quality improvement project: optimising necrotising fasciitis management; evaluation and development of comprehensive guidelines for improved patient outcomes

Ansar M. 1
Inteti K. 2
Kaur S. 2
Mustafai M. 2
Magee C. 2
Wilson J. 2
1 Wirral University Teaching Hospital, Wirral, United Kingdom
2 Wirral university Teaching Hospital, Wirral, United Kingdom

65 An audit of the admissions documentation for patients admitted under the urology service at a district general hospital

Pherwani S.
Rashid H.
Agbugui J.
Lunawat R.
Princess Royal University Hospital, Kings College NHS Foundation Trust, London, United Kingdom

68 Single-centre comparison of clinical practice to the recommendations of the Melanoma Institute of Australia's online prediction tool for sentinel node metastasis in the management of primary melanoma patients

Mills K.
Sweeney K.
NHS Grampian, Aberdeen, United Kingdom

69 An audit to improve effectiveness when booking hand trauma cases via Outlook calendar in a joint orthopaedic and plastic surgery service

Mills K.
NHS Grampian, Aberdeen, United Kingdom

70 Clinical usefulness of medical photography for treating patient with acute wounds

Mills K.
Hill T.
Jivan S.
NHS Mid Yorkshire, Wakefield, United Kingdom

71 How does intra-operative inking of wide local excision margins affect returns to theatre for re-excision?

El-Mekki J. 1
Tsang-Wright F. 1
Wathuge G. 2
Foy V. 2
Nasur A. 1
Farag S. 1
1 Wycombe Breast Unit, High Wycombe, United Kingdom
2 Wycombe Pathology, High Wycombe, United Kingdom

75 A quality improvement project to improve compliance of daily intravenous antibiotic review and documentation during ward rounds

Tan A.S.H.
Ninewells Hospital, Dundee, United Kingdom

81 Osteoporosis prevention in fragility rib fractures in general surgery audit

Dean L.
Browning M.
Bowling K.
Torbay Hospital, Torquay, United Kingdom

89 Improving adherence to NICE guidelines in renal colic management – a closed-loop audit

Mollier J.
Dhillon A.
Mehta S.
Southend University Hospital, Southend-on-Sea, United Kingdom

91 Burns discharge documentation: How to reassure your patient?

Elghouneimy M.
Mclelland T.
Manchester university NHS foundation trust, Manchester, United Kingdom

93 Compliance of emergency operative notes and time taken to operate in cases of appendicectomy per RCS guidelines

Khan M.
Al Qaisi T.
Rotherham NHS Trust, Rotherham, United Kingdom

94 Foundations for foundation – a quality improvement project to develop a pilot surgical skills teaching program

Hill C.
NHS Lothian, Edinburgh, United Kingdom

97 Audit of pacing documentation post-cardiac surgery

Bassi R.
Brazier A.
Binding L.
Barker T.
University Hospital Coventry & Warwickshire NHS Trust, Coventry, United Kingdom

98 Diverticulitis follow-up: are we adherent to ACPGBI guidelines?

Abosheisha M.D.
Shalaby A.
Mudigerenayaz N.
Zarad I.
Shishtawi M.
Attalla R.
Ismaeil M.
Appleton B.
CTM UHB, Bridgend, United Kingdom

104 Management of testicular torsion: A 5-year review of experience in a tertiary hospital in sub-Saharan Africa

Amusat O. 1 2
Fadeyi I. 1
Adekunle I. 1
Tanko J. 1
Farinre O. 1
Adebayo S. 1
1 University College Hospital, Ibadan, Nigeria
2 Luton and Dunstable University Hospital, Luton, United Kingdom

107 Optimising pain management in adult ENT inpatients: a quality improvement audit at Birmingham Heartlands Hospital

Masadeh N. 1
Okoh M. 2
Hirayama Y. 2
Limbrick J. 2
Doshi J. 2
1 Jordan University Hospital, Amman, Jordan
2 Birmingham Heartlands Hospital, Birmingham, United Kingdom

116 The safety and feasibility of day-case ureteroscopy - lessons learnt and future prospective

Manimaran M. 1
Khan H. 2
Krishan A. 1
Dhanasekaran A. 1
1 Sandwell and West Birmingham Hospitals NHS Trust, Birmingham, United Kingdom
2 Queen Elizabeth Hospital, London, United Kingdom

121 Closed-loop audit of the elective colorectal ERAS pathway

Shah M.
Silvestre S.
Nicholson J.
Salford Royal Hospital, Salford, United Kingdom

123 An audit of good surgical practice standards for operative documentation in a mixed EPR and paper system

Koo H.F.
Tan J.
Choudhry M.
Barking Havering Redbridge University Trust Hospital, London, United Kingdom

127 Audit for improvement of surgical operation notes in accordance with the Royal College of Surgeons of England: A closed-loop audit

Al-Gburi S. 1
Alokashi W. 2
Majid M. 3
Safaa M. 3
1 Mosul University, Mosul, Iraq
2 Anbar University, Anbar, Iraq
3 University of Nahrain, Baghdad, Iraq

128 Improving patient outcomes following hip fracture: reducing urinary tract infection rates through timely catheter removal

Amadi-Livingstone C. 1
Mant S. 2
1 York and Scarborough NHS Foundation Trust, York, United Kingdom
2 Royal Devon University Healthcare NHS Foundation Trust, Exeter, United Kingdom

137 A smartphone application to improve junior doctor induction and experience within the trauma department of a major trauma centre: A quality improvement project

Little C. 1 2
Hurst S. 1 3
1 Oxford University Hospitals NHS Foundation Trust, The John Radcliffe Hospital, Oxford, United Kingdom
2 Nuffield Department of Orthopaedics Rheumatology and Musculoskeletal Sciences (NDORMS), Oxford, United Kingdom
3 Oxford University Hospitals NHS Foundation Trust, The Nuffield Orthopaedic Centre, Oxford, United Kingdom

144 Peri-operative good control of HbA1c in known diabetic patients

Younan M.
El Mabara Health Insurance Hospital in Assuit, Assuit, Egypt

145 An audit on general surgical ward round documentation in Royal Blackburn Hospital

Atluri L.M.
Chong K.
Royal Blackburn Hospital, Blackburn, United Kingdom

147 Audit of CT KUB scans: assessing compliance with radiation exposure reduction guidelines

Elamin A. 1
Khalid S.Y. 2
Elamin S. 1
Kee S. 1
1 Sligo University Hospital, Sligo, Ireland
2 St. James's Hospital, Dublin, Ireland

149 Re-assessing adherence to imaging guidelines to minimise the radiation dose in non-contrast CT KUB scans for ureteric colic following local intervention

Jarral F. 1
Abdelrahman H. 2
Mobayen R. 1
Wrazen J. 1
Akbar J. 1
Thomas F. 1
1 Doncaster Royal Infirmary, Doncaster, United Kingdom
2 Doncaster, Doncaster, United Kingdom

154 A bloody and consuming waste: the rate of G&S rejections in trauma patients at level 1 trauma centre

Haq L.
Tailor P.
Shahbaz A.
UHB, Birmingham, United Kingdom

156 Evaluation of three-dimensional digital breast tomosynthesis for intraoperative margin assessment in breast-conserving surgery: preliminary findings

Tamirisa N.
Gianchandani A.
Flores K.
MD Anderson Cancer Center, Houston, USA

160 A closed loop audit to assess prescribing accuracy within a district general hospital in Northern Ireland

Ross A.
Thompson R.
Daisy Hill Hospital, SHSCT, Newry, United Kingdom

161 Bone health in metastatic carcinoma prostate

Sharma G.
Royal Derby Hospital, Derby, United Kingdom

162 Identifying and managing surgical site infections in emergency and elective general surgery: a quality improvement project

Mostafa O. 1
Khan A. 2
Reay M. 1
Kumar P. 1
1 Dudley Group NHS Foundation Trust, Dudley, United Kingdom
2 Sandwell and West Birmingham NHS Foundation Trust, Birmingham, United Kingdom

163 Introduction of a next-day CT KUB pathway for patients with suspected ureteric colic: completion of a closed-loop audit

Simpkins S.
Burton F.
Armstrong K.
Denny F.
Moyes L.
Queen Elizabeth University Hospital, Glasgow, United Kingdom

165 Managing the NELA 'no-lap' cohort: can we do better?

Mostafa O. 1
Khan A. 2
Quddus R. 1
Zaman S. 3
Rajalingam V. 1
1 Dudley Group NHS Foundation Trust, Dudley, United Kingdom
2 Sandwell and West Birmingham Hospitals NHS Trust, Birmingham, United Kingdom
3 University Hospital of Derby and Burton NHS Trust, Burton, United Kingdom

167 Circumcision histology audit

Sharma G.
Royal Derby Hospital, Derby, United Kingdom

168 Introduction of a trial without catheter (TWOC) champion to reduce catheter associated infections in patient having undergone a robotic assisted prostatectomy (RALP) or nephroureterectomy in a district general hospital

Sonanis S.
Stanton N.
Chisvo M.
Royal Blackburn Hospital, Blackburn, United Kingdom

169 Enhancing surgical patient knowledge during discharge: A closed loop quality improvement programme

Agarwal A.
Balasubaramaniam V.
Abdullah N.
Ysbyty Gwynedd, Bangor, United Kingdom

170 Prompt intravenous to oral antibiotic switch (IVOS) in intra-abdominal infections in emergency general surgery

Sajjad M. 1
Tan J. 2
Shahzad K. 1
1 Aintree University Hospital, Liverpool University Foundation Trust, Liverpool, United Kingdom
2 Aintree University Hospital, Liverpool University Foundation Trust, Liverpool, United Kingdom

171 Optimising operating room workflow: a quality improvement project

Elkoumy E. 1
Zahra A. 1
Elgazzar M. 2
Bahget A. 1
1 Souad Kafafi University Hospital Misr University of Science and Technology, Giza, Egypt
2 Desouk General Hospital, Kafr El-Shaikh, Egypt

173 Improving compliance with GIRFT guidelines: A closed loop audit on laparoscopic cholecystectomy operation note documentation at Southend University Hospital

Mollier J.
Abdul-Kader N.
Rosen J.
Syed A.
Southend University Hospital, Southend-on-Sea, United Kingdom

175 Emergency decision-to-incision interval: a multidisciplinary quality improvement project

Elkoumy E.
Ali A.
Sayed M.
Rady A.
Souad Kafafi University Hospital Misr University of Science and Technology, Giza, Egypt

176 Quality improvement in the endoscopic diagnosis of Barrett's oesophagus: enhancing diagnostic accuracy and adherence to guidelines

Dowiedar M.
Albalkiny S.
Gebril M.
Aneurin Bevan University HealthBoard, Newport, United Kingdom

178 Audit on reporting time of mastectomy specimen

Govindasamy V.
Khanagaesparan Thevar T.C.
Tan S.S.
Veenayagam S.
Ng S.W.
Ho J.
James Cook University Hospital, Middlesbrough, United Kingdom

179 Sustainable practices in outpatient hand surgery: an eco-audit of nail bed injury treatments

Glynou S.P. 1 2
Sousi S. 3
Georgiannakis A. 2
Zargaran A. 3 4
Cook H. 4
Ahmed Z. 3
Zargaran D. 3 4
Mosahebi A. 3 4
1 Imperial College London, London, United Kingdom
2 Barts and the London, School of Medicine and Dentistry, London, United Kingdom
3 University College London, London, United Kingdom
4 Royal Free Hospital, London, United Kingdom

180 Timing of commencement of adjuvant therapy following oesophago-gastric cancer resections

Ayantunde B.
Peacock A.
Shenoy S.
Penney N.
Kumar B.
Norfolk and Norwich University Hospital, Norwich, United Kingdom

181 An internal quality improvement project to improve the communication gap between surgeon and patient

Chia E. 1
Tompkins B. 2
Peckham-Cooper A. 1
1 Leeds Teaching Hospitals NHS Trust, Leeds, United Kingdom
2 Betsi Cadwaladr University Health Board, Bodelwyddan, United Kingdom

182 Improving the foundation year doctors’ experience in surgery

Ayantunde B.
Thavayogan R.
Sainz De La Maza Melon A.
Youssef M.
Norfolk and Norwich University Hospital, Norwich, United Kingdom

184 Investigation and outcomes of suspected paediatric appendicitis

El-Gendi A.
Alkistawi F.
Chicken D.W.
Basildon University Hospital, Basildon, United Kingdom

187 Audit of ureteric stone management adherence to NICE and GIRFT guidelines at East Surrey Hospital

Hossain A.
Dhariwal R.
Papikinos P.
East Surrey Hospital, Redhill, United Kingdom

188 Enhancing consent in thoracic surgery: tailored forms for better care

Latif R. 1
AlShammari A. 2
Perikleous P. 2
1 St George's University of London, London, United Kingdom
2 St George's University Hospital, London, United Kingdom

200 Closed cycle audit on length of hospital stay after ACL surgery following GIRFT guidelines

Datta S.
Tahir M.
Akram M.
East Kent University Hospitals NHS Foundation Trust, Margate, United Kingdom

201 A closed loop audit of tranexamic acid use in acute lower gastro-intestinal bleeding

Pherwani S.
Suliman H.
Mardini A.
Hussain K.
Cunin L.
Princess Royal University Hospital, Kings College NHS Trust, London, United Kingdom

202 The importance of gynaecology history taking in surgery: the G-CARES audit.

Mills L. 1
Okocha M. 1
Slim N. 2
Medland V. 1
Maregere E. 3
Aspin C. 3
Vasishat S. 3
Choo Hong Wong M. 3
Chae Y. 3
Khalique H. 3
Herschel P. 3
Huang H.T. 3
Barker L. 3
Lewis E. 3
Anthony Martelock O. 3
Foy G. 3
Bruce C. 3
1 Royal United Hospital, Bath, United Kingdom
2 Gloucester Royal Hospital, Gloucester, United Kingdom
3 Bristol University, Bristol, United Kingdom

203 Extended VTE prophylaxis following lung cancer resections – a clinical audit

Jackson-Smith E.
Qsous G.
Royal Infirmary of Edinburgh, Edinburgh, United Kingdom

204 Fluids prescription in surgical patients – a comparative audit of resuscitation and maintenance practices

Almoshantaf M.B. 1
Barcelona M.D. 2
Sheth H. 1
1 Ealing Hospital, LNWH Trust, London, United Kingdom
2 Imperial College, London, United Kingdom

205 NSAIDs for postoperative analgesia in thoracic surgery – a clinical audit

Jackson-Smith E.
Royal Infirmary of Edinburgh, Edinburgh, United Kingdom

209 Improving timeliness of discharge summaries: evaluating and enhancing compliance with 24-hour GP notification targets

Hanganu C.B.
Cambridge University Hospitals, Cambridge, United Kingdom

211 Evaluating the impact of a new digital platform on surgical handover compliance with Royal College of Surgeons safe handover guidelines

Quimpo J.
Joyner J.
Zhao S.
Dent P.
Vig S.
Croydon University Hospital, London, United Kingdom

214 Assessing pain management in neck of femur fractures: A cross-sectional audit of fascia iliaca blocks

Pattnaik S. 1
Appleton L. 2
Sidhu G.A. 1
Gawad M. 1
Punwar S. 1
1 Lewisham and Greenwich NHS Trust, London, United Kingdom
2 King's College NHS Trust, London, United Kingdom

215 Addressing diagnostic uncertainty in A&E: A study on ankle sprain referrals to trauma clinics

Pattnaik S.
Khalid M.
Kinder I.
Sidhu G.A.
Gawad M.
Punwar S.
Lewisham and Greenwich NHS Trust, London, United Kingdom

216 Biochemical deficiencies in carpal tunnel syndrome: screening before nerve conduction studies

Jelpke E.
Pattnaik S.
Sidhu G.A.
Jeffery M.
Lewisham and Greenwich NHS Trust, London, United Kingdom

217 More is not always merrier in hand surgery patients: venous thromboembolism prophylaxis – are we overprescribing?

Sharma A.
Jose R.
Queen Elizabeth Hospital, University Hospitals Birmingham NHS Foundation Trust, Birmingham, United Kingdom

218 Outcomes of open vs. closed fixation in distal radius fractures: a cross-sectional study

Khalid M.
Pattnaik S.
Ahmed M.
Sidhu G.A.
Gawad M.
Punwar S.
Magan A.
Lewisham and Greenwich NHS Trust, London, United Kingdom

221 Efficacy of gastromiro in small bowel obstruction (SBO): a single-centre experience

Eldoadoa M. 1 2
Khanna A. 1
1 Milton Keyes University Hospital, Milton Keynes, United Kingdom
2 St Helens & Knowsley NHS Teaching Hospital Trust, Merseyside, United Kingdom

223 Audit of day case surgery rates: closed loop audit

Sidhom M. 1 2
Cresswell B. 3
Sundaravadanan S. 3
1 Hampshire Hospitals NHS Foundation Trust, Basingstoke, United Kingdom
2 Kingston Hospitals Foundation Trust, Kingston Upon Thames, United Kingdom
3 Hampshire Hospitals Foundation Trust, Basingstoke, United Kingdom

224 QIP: overview of the current management strategies of diverticular disease in comparison to World Society of Emergency Surgery (WSES) guidelines

Mustafai M.
Aniebo I.
Tarannum T.
Wirral University Teaching Hospital NHS Foundation Trust, Wirral, United Kingdom

226 A retrospective audit of analgesia use in intra-abdominal surgery: a comparison with rectus sheath catheters (RSC) in an ERAS protocol

Patel K.
Southport District General Hospital, Liverpool, United Kingdom

228 Improving patient education in epistaxis management

Ramtohul A.L. 1 2
Brittain R. 1
1 Bedfordshire Hospitals NHS Foundation Trust, Bedford, United Kingdom
2 The Hillingdon Hospitals NHS Foundation Trust, London, United Kingdom

230 Assessing breach clinics at Newham University Hospital as a tool for analysing the effectiveness and efficiency of urological referral pathways

Al-Ghazawi M. 1 2
Saad M. 2
Abay B. 2
Innes-Taylor P. 2
Khan S. 2
1 NHS Greater Glasgow and Clyde, Glasgow, United Kingdom
2 Barts NHS Health, London, United Kingdom

232 The Lister experience: an audit of the correlation between MRI PI-RADs 2 and 3 lesions and clinically significant prostate cancer (csPSC) on trans-perineal prostate biopsy

Kucheria A.
Alomari M.
Benabdulla N.
Shaikh Hasan A.
Rashid S.
Farahani D.
Lott S.
Hosny M.
Lister Hospital, Stevenage, United Kingdom

236 Appendicitis management and outcome for children and young people in Milton Keynes University Hospital

Yousif E.
MKUH, Milton Keynes, United Kingdom

237 A quality improvement initiative to improve prescription compliance of routine post-operative drugs in thoracic surgery

Pratik P.I.P.
Dhanji A.R.
Patel K.
St Bartholomew's Hospital, London, United Kingdom

239 Optimising electronic records for urology handover: a comparative audit

Asfour A. 1
Shurrab M. 1
Lubbad H. 2
1 UHL NHS Trust, Leicester, United Kingdom
2 University of Leicester, Leicester, United Kingdom

241 Evaluating histology result documentation in general outpatient clinic letters at Ealing Hospital

Abu A.
Almoshantaf M.B.
Abdalla S.
Ealing Hospital, London, United Kingdom

242 Intravenous fluid therapy for the acute general surgical patient

Okoh M.
Sabbubeh B.
Gates Z.
Bedford M.
University Hospitals Birmingham, Birmingham, United Kingdom

244 Assessment of the compliance of CTPA requests to standard guidelines and PE detection rate (re-audit)

Hamdy A. 1
Jarral F. 1
Imam S. 2
1 Doncaster and Bassetlaw Teaching Hospital NHS trust, Doncaster, United Kingdom
2 University of Sheffield, Sheffield, United Kingdom

246 Diabetic foot ulcer pathway for the emergency department

Silva Torres J.A.
Taylor J.
Vig S.
Solanki P.
Croydon University Hospital, London, United Kingdom

248 Plastic surgery antimicrobial guidelines quality improvement project and compliance audit

Majeed R.
Gakpetor D.
Salloum N.
Waterston S.
Ninewells Hospital, Dundee, United Kingdom

256 Clinical audit of transurethral resection of bladder lesions performed in Letterkenny University Hospital

Khalid S.Y. 1 2
Waraich T.A. 2
Muddassir S.M. 2
1 St. James's Hospital, Dublin, Ireland
2 Letterkenny University Hospital, Letterkenny, Ireland

261 Prostate cancer patient support audit

Iftikhar U.
Low Z.Y.
Dias M.
Nunow M.
Chakravarti S.
Pina S.
John B.
Croydon Health Service, London, United Kingdom

262 Audit of out-patient clinic letter: writing directly to patient

Dey S.
de Courcey C.
Hardwicke J.
University Hospital Coventry & Warwickshire, Coventry, United Kingdom

264 Improving the antibiotic management of patients with epididymo-orchitis at a local NHS general hospital

Chataway J.
O'Donnell C.
Li C.Y.
Barts Health NHS Trust, London, United Kingdom

267 The hidden crisis of surgical site infections in Yemen: the first audit of incidence and protocol compliance in two major public hospitals in Sana'a

Al-Eryani F.
Al-Shaikh B.
Al-Nofish F.
Al-Tegani A.
Al-eryani B.
Alshargabi H.
Alamri Y.
Al-Shehari M.
Faculty of Medicine, Sana'a of University, Sana'a, Yemen

268 An audit on epistaxis management and the potential role of ambulatory care in epistaxis patients

Kingue Sousseing M.
Chandegra R.
Wakeford W.
Sood T.
Luton & Dunstable University Hospital, Luton, United Kingdom

273 Evaluating compliance of digitalised general surgery operation notes with RCS England guidelines: A clinical audit

Ooi S.Z.Y.
Al-Sarireh B.
Department of Surgery, Morriston Hospital, Swansea, United Kingdom

274 Achieving the hip fracture best practice tariff by enhancing delirium assessment during clerking

Linn H.H. 1
Moosa Al. 2
Yarlagadda R. 2
Davies J. 2
1 Imperial College Healthcare NHS Trust, London, United Kingdom
2 University Hospitals Plymouth NHS Trust, Plymouth, United Kingdom

276 Improving perioperative documentation of microvascular breast free flaps for enhanced outcomes: A closed-loop audit

Frommer M.L. 1 2
Brosnan J. 1
Karoshi A. 3
Awad L. 1 2
Langridge B. 1 2
Nikkhah D. 1 2
Hamilton S. 1
1 Department of Plastic Surgery, Royal Free Hospital, London, United Kingdom
2 Division of Surgery and Interventional Science, University College London, London, United Kingdom
3 Division of Medicine, University College London, London, United Kingdom

277 Ten-year follow up of diabetes mellitus remission following bariatric surgery: a single surgeon experience

Abdelrahim A.
Amr B.
Yusuf Y.
Mitchell A.
Darlington Memorial Hospital, Darlington, United Kingdom

278 An audit of the use of primary ureteroscopy in the management of acute symptomatic ureteric stones: a district hospital experience

Abdulrasheed H. 1
Adenipekun O. 2
1 Birmingham Heartland Hospital, University Hospital NHS Foundation Trust, Birmingham, United Kingdom
2 Surgery Interest Group in Africa, Lagos, Nigeria

283 Audit of management of acute scrotal pain presentation at a district hospital

Abdulrasheed H.
Adenipekun A.
Ho K.
Birmingham Heartland Hospital, University Hospital Birmingham NHS Trust, Birmingham, United Kingdom

289 Assessing the diagnostic quality of chest x-rays in a district general hospital

Otote J.
Ravindran D.
Nijjar R.
Niranjan K.
Barking Havering and Redbridge University Hospitals Trust, London, United Kingdom

292 Upper limb soft tissue – is one-stop ultrasound better?

Birkenhead P.
Aamir J.
Gunn C.
Stevenson H.
Chan D.
Liverpool University Hospital Foundation Trust, Liverpool, United Kingdom

293 Redefining recovery: the safety and efficacy of day case surgery in immediate implant-based breast reconstruction

Shahrokhi N. 1
Joshi Puthur S. 2
Itaman U. 3
Karas A. 2
Johnpulle M. 2
Nasir N. 2
1 Pulvertaft Centre, Royal Derby Hospital, Derby, United Kingdom
2 Department of Breast Surgery, North Manchester General Hospital, Manchester, United Kingdom
3 Department of General Surgery, North Manchester General Hospital, Manchester, United Kingdom

294 A closed loop audit examining the use of a technology-enhanced ward round template to improve surgical ward round documentation

D'Souza F.
Dheden N.
Moore J.
Dawas K.
University College London Hospital, London, United Kingdom

297 An audit of correlation between trans-perineal prostate biopsies and prostatectomy histology for Gleason 6 prostate cancer

Kucheria A.
Warner R.
Lister Hospital, Stevenage, United Kingdom

298 Breast surgery documentation: a retrospective audit of compliance with Association of Breast Surgery operation note guidelines

Riad A.
Stoner R.
Khan A.
West Middlesex University Hospital, London, United Kingdom

301 Adherence to ACPGBI guidelines for management of pseudo-obstruction

Khattak M.A.
Shah M.
Stamatiou D.
Heartlands Hospital, University Hospitals Birmingham NHS Foundation Trust, Birmingham, United Kingdom

302 Setting the gold standard: improving the reliability of follow up after orthopaedic admission at a tertiary trauma centre

Choy B.
Tang E.J.L.
Hrycaiczuk A.
Spencer S.
Queen Elizabeth University Hospital, Glasgow, United Kingdom

304 QIP: To improve the attendance of the lunch and learn sessions in general surgery

Jawad H.A.
Bashir N.
Basildon University Hospital, Essex, United Kingdom

306 Abandoned Whipples procedure

Meher S.
Kings College Hospital, London, United Kingdom

307 Enhancing primary care practices quality improvement: FIT testing in the presence of rectal bleeding

Verani L.
Yahia Z.
Wigan Wrightington and Leigh, Wigan, United Kingdom

311 Assessment of TWIST score utilisation and documentation in the diagnosis of testicular torsion: a retrospective study across a multi-site trust

Chkir B.
Butt M.F.
Saqib M.
Furness General Hospital, Barrow-in-Furness, United Kingdom

313 Implementing resuscitation huddles: enhancing teamwork and patient safety in emergency response calls

Quimpo J.
Spellman-Burch H.
Archer A.
Croydon University Hospital, London, United Kingdom

314 Evaluating compliance with NICE standards for gallstone disease management: a retrospective study at a district general hospital

Chkir B.
Tabaie E.
Patel P.
Furness General Hospital, Barrow-In-Furness, United Kingdom

316 Improving adherence to renal colic referral pathways in a DGH: A two-cycle clinical audit

Mohamed A.
Alkaseek A.
Ibrahim M.
Bhardwa J.
Wexham Park Hospital, Slough, United Kingdom

322 Improving breast surgery departmental induction

Berezowska A.A. 1
Patel R. 2
Pillai G. 2
1 Bedford Hospital, Bedford, United Kingdom
2 West Middlesex University Hospital, London, United Kingdom

325 A comprehensive audit on the diagnosis, management and follow-up of bladder cancer in a tertiary hospital

Carvalho M. 1
Munier L. 2
Curry D. 1
1 Belfast Health and Social Care Trust, Belfast, United Kingdom
2 Queen's University Belfast, Belfast, United Kingdom

326 A retrospective audit assessing the diagnostic yield of barium swallow in patients with suspected upper GI cancer

Madasu A. 1
Bhardwaj A. 2
Vinayagam R. 3
1 Calderdale and Huddersfield NHS Foundation Trust, Halifax, United Kingdom
2 Harrogate and District NHS Foundation Trust, Harrogate, United Kingdom
3 Bradford Teaching Hospitals NHS Foundation Trust, Bradford, United Kingdom

327 Studying the use of MRI and ultrasonography in patients between the ages 40 to 60 years old following glenohumeral dislocations

Moss M.
Sabharwal S.
Durand-Hill M.
Sellahewa S.
St Mary's Hospital, London, United Kingdom

328 Clinical audit on compliance with NELA-predicted mortality rate documentation on surgical consent forms

Kuttuva S.
Jones E.
Kasireddy S.
Box B.
Northumbria Healthcare NHS Foundation Trust, North Tyneside, United Kingdom

329 Administration of fascia-iliaca blocks for hip fracture patients on admission to the A&E department at Southport DGH

Moss M.
Sha S.
Sangani C.
Gledhill P.
Southport Hospital, Southport, United Kingdom

331 Automated ureteric stent register using Microsoft 365 lists

Latham J.
Mohamed W.
Royal Glamorgan Hospital, Cardiff, United Kingdom

332 Surgical site infection in living donor nephrectomy

Yusuf Z.
Knight S.
Queen Elizabeth University Hospital, Glasgow, United Kingdom

333 Cracking under pressure: assessing rib fracture care

Hussain K.
Suliman H.
Ahmed R.
Dudek J.
Singhal T.
Princess Royal University Hospital, King's College Hospital NHS Foundation Trust, London, United Kingdom

335 Evaluating outcomes, safety, and antibiotic use in precision point transperineal prostate biopsy: insights from a 300-patient audit

Desouky O.
Faty M.
Campbell I.
East Lancashire Hospitals NHS Trust, Blackburn, United Kingdom

337 Negative rate of appendicectomy in the paediatric population – time for drastic changes

Karagiannidis G. 1
Pervez A. 2
1 Imperial College London, London, United Kingdom
2 Ipswich Hospital, Ipswich, United Kingdom

338 Pilot study introducing procedure-specific consent forms for laparoscopic cholecystectomy to improve consent form compliance

Gardner S.
Duck E.
Darke E.
North Bristol Trust, Bristol, United Kingdom

342 Recognition and management of actively dying patients in surgery

Fowler C.
McSorely M.
Royal Infirmary Edinburgh, Edinburgh, United Kingdom

345 Evaluating preoperative urine culture’s in endourology surgeries

Challapalli P. 1
Prabaharan P. 1
Thompson W. 2
1 Royal Liverpool University Hospital, Liverpool, United Kingdom
2 Royal Liverpool University Hospital, Royal Liverpool University Hospital, United Kingdom

348 Audit of timed diagnostic pathway adherence for suspected prostate cancer

Fazili A.
Godil F.
Shahid J.
Bedfordshire Hospital, Bedford, United Kingdom

350 Modernising communication in the ENT outpatient setting

Middleton A.L.
Great Western Hospital, Swindon, United Kingdom

356 Timing of surgical fixation of distal radius fracture: A district hospital fixation

Dahal B.
Yeoh C.J.C.
Lister Hospital East and North Hertfordshire NHS Trust, Stevenage, United Kingdom

357 Quality improvement project on uroflowmetry in urology outpatient clinic

Carvalho M.
Belfast Health and Social Care Trust, Belfast, United Kingdom

358 Updating the general surgery induction booklet using national guidelines and feedback: a quality improvement project

Brennan M.
Sharif M.
Yadu S.
Davies J.
Taylor F.
Joseph J.
Whipps Cross Hospital, London, United Kingdom

362 Evaluating surgical nurses' knowledge of elective colorectal surgery enhanced recovery after surgery (ERAS)

Fatim S.
Husain S.
Kaur H.
Fawole A.
Pinderfields Hospital, Wakefield, United Kingdom

365 Evaluating the efficacy of hyperbaric oxygen therapy as an adjuvant therapy in the management of diabetic foot ulcers: a retrospective audit

Madasu A. 1
Bhardwaj A. 2
Arias Chavez J. 3
Al-Natsheh W. 4
1 Calderdale and Huddersfield NHS Foundation Trust, Halifax, United Kingdom
2 Harrogate and District NHS Foundation Trust, Harrogate, United Kingdom
3 Nottingham University Hospitals NHS Trust, Nottingham, United Kingdom
4 Manchester University NHS Foundation Trust, Manchester, United Kingdom

366 Do surgical patients know their consultant? An audit based on the Francis report

Thorne T. 1 2
McCabe J. 1 2
Littler S. 1
Nandra S. 1 2
Akbari K. 1
1 University Hospitals Coventry and Warwickshire, Coventry, United Kingdom
2 University of Warwick, Coventry, United Kingdom

367 Escalation and Do Not Resuscitate status in surgical patients: a quality improvement project

Lagunathan S.
MSE, Southend, United Kingdom

370 Urology day case procedure, are we achieving patient satisfaction?

Islam F.
Oder R.
Patel K.
Royal Derby Hospital, Derby, United Kingdom

371 Quality improvement project on head injury and driving advice

Carvalho M.
McGuigan L.
Northern Health and Social Care Trust, Coleraine, United Kingdom

372 Evaluation of outcome of day case cholecystectomies

Bunga M.S. 1
Taggarsi M. 2
Nagaraju V. 3
Mothe B.S. 4
1 Stepping Hill Hospital, Manchester, United Kingdom
2 George Elliot Hospital, Nuneaton, United Kingdom
3 Wigan NHS Foundation Trust, Manchester, United Kingdom
4 Stoke on Trent NHS Foundation Trust, Manchester, United Kingdom

374 A retrospective study of pre-operative fasting times of CEPOD list patients in a district hospital: lessons to be learned to minimize the fasting time

Sikder D.K.
Ibrahim B.
Mansour M.
North Manchester General Hospital, Manchester, United Kingdom

377 SSPAM: surgical safe prescribing with anthropomorphic measurements

Mills L.
Okocha M.
Royal United Hospital, Bath, United Kingdom

382 An audit of the use of the Modified Glasgow Score in admissions of acute pancreatitis at a district general hospital

Walsh J.
Asif H.
East Sussex NHS Trust, Hastings, United Kingdom

383 Management and outcomes of small bowel obstruction- not just the ‘now’ but what about the ‘next’

Inteti K.
Magee C.
Wilson
Babiker M.
Rashid R.
Wirral University Hospitals NHS Foundation Trust, Merseyside, United Kingdom

384 Improving referrals from general surgery to ageing and complex

Price S.
Alićehajić-Bečić D.
Wrightington, Wigan and Leigh NHS Foundation Trust, Wigan, United Kingdom

386 Evaluation of adherence to updated nice guidelines for paediatric grommet insertion: A retrospective study

Fatima S.
Dhillon D.S.
Pinderfields Hospital, Wakefield, United Kingdom

387 An audit of timing of cholecystectomy for patients admitted with acute cholecystitis in a district general hospital

Selva Raj D.R.
Balakrishnan K.R.
Sabanathan S.
Department of General Surgery, Wrexham Maelor Hospital, Wrexham, United Kingdom

393 Quality improvement project: would a virtual departmental tour be a useful adjunct to traditional face to face induction?

Chana N.
Win N.
Mills K.
Jivan S.
Pinderfields General Hospital, Wakefield, United Kingdom

394 Reducing length of stay after appendicectomy for acute appendicitis: A quality improvement project

Mondragon M.
Karmarkar R.
Royal Wolverhampton NHS Trust, Wolverhampton, United Kingdom

395 Optimising surgical training: a comparative survey-based study of cutting-edge Evelina day surgery unit (DSU) and main theatre efficiency and outcomes

Khan S.
Ardani S.
Kolaityte V.
Filson S.
Guy's and St Thomas' NHS Foundation Trust, London, United Kingdom

397 Project 3.0, high-volume theatre lists for low-complexity urological procedures

Rasheed M.
Khaled G.
Kevin G.
Mazharuddin S.
Flaviu B.
Queen's Hospital, Romford, United Kingdom

399 The use of British Society of Gastroenterology management of lower GI bleeds algorithm in clinical practice to stratify admissions and discharge of patients

Walsh J.
Asif H.
East Sussex NHS Trust, Hastings, United Kingdom

401 Improving assessment of plastic surgery patients at a level 1 major trauma centre

Theocharidou L.
Pang C.
Loh C.
Cambridge University Hospitals, Cambridge, United Kingdom

408 Management of gall-bladder polyps in an NHS hospitals trust in London: an audit

Naseem S.
Dornseifer M.D.
Zafar S.
Shaik M.
Rajagopal P.
Osilli D.
Barking, Havering & Redbridge University Hospitals NHS Trust, Romford, United Kingdom

409 ISCP – can we help trainees navigate their portfolios?

Chana N.
Jivan S.
Vaughan-Williams S.
Mistry D.
Pinderfields General Hospital, Wakefield, United Kingdom

413 Comparing outcomes of lower ureter management in nephroureterectomy

Desouky O.
East Lancashire Hospitals NHS Trust, Blackburn, United Kingdom

423 Compliance with completion of organ donor checklist form and ABO crossmatch safety check pre-renal transplant surgery

Bholah Z.
Manchester Royal Infirmary, Manchester, United Kingdom

424 Impact of ERAS ward round template: A closed-loop audit on enhancing post-operative recovery in colorectal surgery

Alkaseek A.
Ahmed H.
Oyeniyi S.
Ahmed M.
Mohamed A.
Salerno G.
Wexham Park Hospital, Wexham, United Kingdom

429 Assessing choice of intertrochanteric hip fracture intervention compared to fracture pattern and classification, a closed cycle audit

Esworthy G.
Gao H.C.K.
Akhtar M.
Sharma V.
Lincoln County Hospital, Lincoln, United Kingdom

430 Quality improvement project: would a virtual departmental tour be a useful adjunct to traditional face-to-face induction?

Mustafa A.
Win N.
Chana N.
Jivan S.
Mid Yorkshire Teaching NHS Trust, Wakefield, United Kingdom

432 Audit about IV fluid management in NBM surgical patients

Zehra B.
Siddique M.H.
Muhamma M.
Khan A.A.
Halmey S.
Good Hope Hospital, Birmingham, United Kingdom

435 Simple intervention to save a limb: improving the quality of discharge summaries for vascular surgery inpatients

Wijaya A.
Claxton M.
Constantinou C.
Kiernan-O’Donnell R.
Skerett B.
Khalaf H.
Russell J.
Musgrove Park Hospital, Taunton, United Kingdom

440 Is PSMA PET scan data valuable for recurrence of PSA rise in patients after RARP?

Joseph J.
Desai C.
University Hospitals Coventry & Warwickshire NHS Trust, Coventry, United Kingdom

443 Cutting through the details: an audit of surgical note quality in emergency general surgery

Liyanage D.
Queen Elizabeth Hospital, Birmingham, United Kingdom

445 Direct adult tonsillectomy pathway: an evaluation of impact on waiting times and outcomes in an Irish tertiary otorhinolaryngology department

Shomoye D. 1 2
Naude A. 1 2
1 Beaumont Hospital, Dublin, Ireland
2 Royal College of Surgeons in Ireland, Dublin, Ireland

449 Closed loop audit of nasal trauma management and introduction of opt-in system for patient follow up

Kruczynska A.
Cain A.
Raigmore Hospital, NHS Highlands, Inverness, United Kingdom

451 Around the clock: is there a difference in liver transplants during the day-time vs night-time?

Liyanage D.
Queen Elizabeth Hospital, Birmingham, United Kingdom

452 Reducing patient waiting times in fracture clinic by changing X-ray flow: a quality improvement project

Atkins B.
Barnard T.
Sukirthan S.
Northwick Park Hospital, London, United Kingdom

455 Bridging the gap: enhancing outcomes in neck of femur fracture care through optimized handover between orthopaedic and ortho-geriatric teams

Abu Hussein B.
Khalaf A.
Tilley S.
Bilgrami T.
University Hospital Southampton, Southampton, United Kingdom

458 Suspected cauda equina syndrome: audit of compliance to current local pathway

Maxwell M.
Mceneaney L.
Maheswaran A.
Zhang J.
Khan I.
ElNawasany M.
Abdel-Hafez Y.
Day A.M.
Hulme C.
Epsom and St Helier University Hospitals NHS Trust, Carshalton, United Kingdom

461 Admission patterns of elderly patients with head injuries in acute surgical unit

Naveed S.
Basildon University Hospital, Basildon, United Kingdom

465 New patient fracture clinic referrals and discharges: a quality improvement project at King's Mill Hospital

Naccarato E.
Kumaran S.
Awarah H.
Eley C.
Dili L.
Lawton G.
Salem-Saqer H.
Mitra A.
Sherwood Forest Hospitals, Sutton-in-Ashfield, United Kingdom

467 Evaluating and enhancing the quality of record keeping in a single surgical: A closed loop clinical audit using the surgical tool for auditing records (STAR) scoring system

Abdul Mubarack O.M.O. 1 2
Wickramasinghe D. 1
Wickramasinghe D. 1
1 National Hospital of Sri Lanka, Colombo, Colombo, Sri Lanka
2 Colchester General Hospital, Colchester, United Kingdom

473 What is the real cost of delay in incision and drainage of abscesses in the NHS?

Joseph J.
Linder C.
University Hospitals Coventry, Coventry, United Kingdom

477 Improving cholecystitis care in general surgery: A closed loop audit on emergency cholecystectomy pathway

Poomalai I. 1
George J. 2
1 Kongunad Hospitals Pvt Ltd, Coimbatore, India
2 University Hospitals of Leicester NHS Trust, Leicester, United Kingdom

484 Exploring the national joint registry data collection: learning from an audit at a London-based district general hospital

Kumar A. 1
Khajuria A. 1
Kharma N. 1
Tannirandorn P. 2
Yong C. 2
Ibrahim M. 1
1 Whittington Health NHS Trust, London, United Kingdom
2 University College London Medical School, London, United Kingdom

485 Managing epistaxis, do we know it all?

Gurung G.
Amatya A.
Bakare O.
Rai P.
Landolfi C.
Hereford County Hospital, Hereford, United Kingdom

487 Improving post-operative X-ray documentation in patients with femoral fractures

Prithviraj D.
Hosny S.
Wexham Park Hospital, London, United Kingdom

489 An EPIC takeover of ENCOMPASS – evaluation of medical note-taking and patient safety

Jaffer M.
Eves P.
Royal Victoria Hospital, Belfast, United Kingdom

491 Pain with no gain? An audit on nephrostomy insertion for non-urological malignancies

Hajuthman W.
Batson-Oatel S.
Phillips A.
Saji E.
Ponsailapathi N.
Vinayagam S.
Pai A.
Northampton General Hospital, Northampton, United Kingdom

492 Indocyanine green (ICG) vs lymphoscintigraphy for SLNB in melanoma

Sweeney K. 1
Rahman K. 2
1 Aberdeen Royal Infirmary, Glasgow, United Kingdom
2 Aberdeen Royal Infirmary, Aberdeen, United Kingdom

494 Post peripheral angioplasty assessment: A single unit experience

Jamil S.
Naseer A.
Elkorety M.
Sarin S.
West Hertfordshire Trust, Watford, United Kingdom

Subjects: Robotics and Digital Surgery

54 Using a modified Delphi process to explore international surgeon-reported benefits of robotic-assisted surgery to perform abdominal rectopexy

Keating T. 1
Fleming C. 2
Nally D. 1
Brannigan A. 1
1 Mater Misericordiae Hospital, Dublin, Ireland
2 University Hospital Limerick, Limerick, Ireland

61 Assessment of the ‘myrecovery’ app in supporting patients undergoing elective orthopaedic surgery

Starkey A.
Johnson D.
Stockport NHS Foundation Trust, Stockport, United Kingdom

118 Integration of robotics in minimally invasive neurosurgery: advancements and outcomes

Oyoyo H.
Adegoke T.
Elam C.
College Of Medicine University of Ibadan, Ibadan, Nigeria

134 A comparison of digital templating with and without scalers in total knee arthroplasty

Arain Z.
Durand-Hill M.
Murphy J.
Northwick Park Hospital, London, United Kingdom

155 Robotic segmental resection of splenic flexure tumour and intracorporal anastomosis with ICG injection

Dadigamuwage S.
Alwis M.
Rajesh T.K.
University Hospitals Plymouth NHS Trust, Plymouth, United Kingdom

157 Barriers and recommendations for the implementation of robot-assisted minimally invasive surgery in Africa

Falola A. 1
Das U. 2
Singh S. 2
Oluwagbemi A. 3
Etta R. 1
1 University of Ibadan College of Medicine, Ibadan, Nigeria
2 Muzaffarnagar Medical College, Bahadarpur, India
3 Marigold Hospital and Critical Care Center, Lagos, Nigeria

210 Haemostatic adjuncts in TORS: A systematic review and cohort study

Sattar Z.
Watts E.
Ahsan S.F.
Royal Wolverhampton NHS Trust, Wolverhampton, United Kingdom

272 Robotic Hartmann's colectomy for complex perforated acute diverticulitis: a case report

Hatem F. 1
Fenech J. 2
1 Royal Victoria Infirmary, Newcastle, United Kingdom
2 St Thomas Hospital, London, United Kingdom

308 Post-operative CRP profiles differ between laparoscopic vs robotic-assisted surgery for colorectal cancer: a tertiary centre experience

Georgopoulou N.
Shadbolt E.
Fitton C.
Miller J.
Campbell K.
Shah A.
Ninewells Hospital, Dundee, United Kingdom

410 Service evaluation of the CMR Versius® surgical robot in Wales

Leveridge B. 1
Eley C. 1 2
Ansell J. 2
Torkington J. 2
Sogaolu O. 2
McKenna M. 2
Sharma A. 2
Botros M. 2
Peevor R. 3
Jones R. 3
Butterworth C. 3
Rehman S. 3
1 Cardiff University School of Medicine, Cardiff, United Kingdom
2 University Hospital of Wales, Cardiff, United Kingdom
3 Ysbyty Gwynedd Hospital, Bangor, United Kingdom

447 How robotic platforms are revolutionizing colorectal surgery techniques: a comparative review

Hussain M. 1
Jaffar-Karballai M. 2
Kayali F. 3
Bashir M. 4
Murtada A. 5
1 Hull York Medical School, York, United Kingdom
2 Chelsea and Westminster NHS Trust, London, United Kingdom
3 Royal Liverpool University Hospital Trust, Liverpool, United Kingdom
4 Neurovascular Research Laboratory, Faculty of Life Sciences and Education, Cardiff, United Kingdom
5 Betsi Cadwaladr University Health Board-Department of General Surgery, Bodelwyddan, United Kingdom

Subjects: Systematic Review and Meta-Analyses

19 The role of antibiotics in preventing pancreatitis in patients with obstructive jaundice and undergoing endoscopic retrograde cholangiopancreatography

Asogwa S. 1
Uwumiro F. 2
Alemenzohu H. 3
Alemenzohu H. 4
1 London North West University Healthcare NHS Foundation, London, United Kingdom
2 University of Jos College of Health Sciences, Jos, Nigeria
3 Jackson State University, General Surgery, Lynch St. Jackson MS- USA, Jackson, USA
4 St Barnabas Hospital, Hepatobiliary Surgery, New York, USA

26 Systematic review and meta-analysis of the long-term laryngeal function and quality of life following treatment of early glottic cancer

Qasem M. 1
Qasem N. 2
Milinis K. 1
Kinshuck A. 1
1 Liverpool University Hospitals Foundation Trust, Liverpool, United Kingdom
2 Jordan University of Science and Technology, Irbid, Jordan

27 Assessing bone quality and formation with osteoblasts: A systematic review of characterisation approaches

Varghese T.
UCL, London, United Kingdom

62 The perioperative outcomes of robotic radical cystectomy versus non-robotic radical cystectomy for muscle-invasive bladder cancer and high-risk non-muscle-invasive bladder cancer

Sanni Q. 1
Geirbely A. 2
Alzamzami M. 3
Alhassan A. 2
Elnaeem K. 4
Alhassan E. 5
Anderson J. 3
1 South Warwickshire University NHS Foundation Trust, Warwick, United Kingdom
2 Beaumont Hospital, Dublin, Ireland
3 South Warwickshire University NHS Foundation Trust, Warwick, United Kingdom
4 Our Lady's of Lourds Hospital, Dublin, Ireland
5 Omdurman Teaching Hospital, Khartoum, Sudan

72 Fertility preservation in neuro-oncology patients: what are the options?

Osborne-Grinter M. 1
Sanghera J. 2
Chiamaka Bianca O. 3
Kaliaperumal C. 4
1 Centre for Surgical Research, Bristol, United Kingdom
2 College of Medicine, Edinburgh, United Kingdom
3 College of Medicine, Ibadan, Nigeria
4 Centre for Clinical Brain Science, Edinburgh, United Kingdom

86 The efficacy of the transversus abdominis plane block in abdominoplasty: A systematic review and meta-analysis

Taha N. 1
Hodson L. 1
Tong K. 2
Zahari F. 1
Hoo Z.L. 3
Wong Y.W. 4
Rahman S. 1
1 Leeds General Infirmary, Leeds, United Kingdom
2 Queen Victoria Hospital, East Grinstead, United Kingdom
3 Harrogate District Hospital, Harrogate, United Kingdom
4 St James University Hospital, Leeds, United Kingdom

92 Comparison of 4DryField PH vs Seprafilm in reducing incidence of adhesion and preventing ASBO following abdominopelvic surgery: a systematic review

Haji Cassim S.
Karim R.
Patel B.
Barts Cancer Institute, London, United Kingdom

102 Telemedicine in Brazil – a systematic review of systematic reviews published in the last 10 years

Mineli de Lima T.
Verbicaro Perdomo C.
Namie Matie S.
Nunes Bessane T.
Centro Universitário de Jaguariúna, Jaguariúna, Brazil

113 Effects of statin therapy on patients with coronary artery bypass surgery: a systematic review and meta-analysis of randomized controlled trials

Ramadan M. 1
Bashour G. 2 3
Eldokmery E. 4
Dway A. 5
Alsalhi K. 6
Emad A. 7
Labieb F. 8
1 Faculty of Medicine, Suez University, Suez, Egypt
2 Faculty of Medicine, Tishreen University, Latakia, Syrian Arab Republic
3 Cancer Research Center, Tishreen University Hospital, Latakia, Syrian Arab Republic
4 Faculty of Medicine, Benha University, Qalyubia, Egypt
5 Faculty of Medicine, AL Andalus University, Tartus, Syrian Arab Republic
6 Faculty of Medicine, Batterjee medical college, Jeddah, Saudi Arabia
7 Faculty of Medicine, Zagazig University, Sharqiyah, Egypt
8 Faculty of Medicine, Beni-Suef Univesity, Beni-Suef, Egypt

115 Efficacy and safety of combined carbazochrome sodium sulphate and tranexamic acid for blood loss control in patient with total hip arthroplasty

Elghouneimy M. 1
Wahb M. 2
Khater M. 3
Qandeel M. 4
Elrefae A. 4
Seif A. 5
Amgad M. 5
Ahmed O. 5
Mashhour M. 6
1 Manchester university NHS foundation trust, Manchester, United Kingdom
2 Kettering general hospital NHS foundation trust, Kettering, United Kingdom
3 Diana princess of Wales hospital, Grimsby, United Kingdom
4 Northwick Park Hospital, London, United Kingdom
5 Faculty of Medicine, Azhar University, Cairo, Egypt
6 Benha Insurance Hospital, Benha, Egypt

136 Falciformopexy as an alternative to omentopexy in perforated peptic ulcer repair: a systematic review and meta-analysis

Abosheisha M.D. 1
Shalaby A. 1
Zarad I. 1
Nasr E. 2
Abdelsalam M. 1
1 CTM UHB, Bridgend, United Kingdom
2 UHBW NHS Trust, Bristol, United Kingdom

139 Enhancing case-based learning in medical education: a systematic review of extended reality applications and their impact on student engagement and learning outcomes

Thileepan L. 1
Suhail S. 2
Patel A. 1
1 UCL, London, United Kingdom
2 Royal Free NHS Trust, London, United Kingdom

152 The use of radiological markers in hip templating: systematic review and meta-analysis

Hutchinson K. 1
Syed L. 2
Musbahi O. 3
1 Croydon NHS trust, London, United Kingdom
2 University Hospitals Sussex NHS Foundation Trust, Brighton, United Kingdom
3 Imperial College London, London, United Kingdom

196 Custom triflange acetabular components in revision total hip arthroplasty: a systematic review and meta-analysis

Tahir M.
Datta S.
Akram M.
Arealis G.
East Kent University Hospitals NHS Foundation Trust, Margate, United Kingdom

197 Comparative analysis of medial knee pivot vs post stabilised knee implants in patients undergoing total knee arthroplasty (TKA): a systematic review and meta-analysis

Tahir M.
Datta S.
Akram M.
Arealis G.
East Kent University Hospitals NHS Foundation Trust, Margate, United Kingdom

227 Safety of mesh repair in strangulated groin hernias: a systematic review and meta-analysis

Ekwesianya A.C. 1
Ayantunde A. 1
Nour H. 2
1 Southend University Hospital, Westcliff-on-Sea, United Kingdom
2 Royal Lancaster Infirmary, Lancaster, United Kingdom

249 Systematic review on isolated nailbed injuries and management

Theocharidou L.
CUH, Cambridge, United Kingdom

288 Real world sex differences in ascending aortic aneurysm surgery. a comprehensive meta-analysis

Alroobi A. 1
Al-Tawil M. 2 3
Geragotellis A. 4
Ghaben N. 3
AboAbdo M. 1
Alaila D. 1
Sulaiman W. 5
Salim H. 4
Leick J. 2
Almaghrabi S. 6
Haneya A. 2
1 Faculty of Medicine, Islamic University of Gaza, Gaza, Palestine
2 Department of Cardiac and Thoracic Surgery, Trier Heart Centre, Trier, Germany
3 Faculty of Medicine, Al-Quds University, East Jerusalem, Palestine
4 Faculty of Health Sciences, University of Cape Town, Observatory, South Africa
5 Faculty of Dentistry, Al-Azhar University, Gaza, Palestine
6 Department of Cardiology, Maria-Hilf Hospital Daun, Daun, Germany

295 Evaluating the psychometric performance of QuickDASH for upper limb patient-reported outcomes

Elhariry M. 1
Khaleeq T. 1
Munigangaiah S. 2
1 Sandwell General Hospital, Birmingham, United Kingdom
2 Alder Hey Children's Hospital, Liverpool, United Kingdom

318 Laser lipolysis with and without suction on arm circumference: a systematic review and meta-analysis

Wong Z.Y. 1
Murugan V. 2
Faderani R. 3
Kanapathy M. 3
Mosahebi A. 3
1 Morriston Hospital, Swansea, United Kingdom
2 Queens Medical Centre, Nottingham, United Kingdom
3 UCL, London, United Kingdom

319 Does colour matter? A systematic review of colour match assessment and innovation in skin flaps and skin grafts

Wong Z.Y. 1
Ou K.Q. 2
Faderani R. 3
Kanapathy M. 3
Mosahebi A. 3
1 Morriston Hospital, Swansea, United Kingdom
2 Southmead Hospital, Bristol, United Kingdom
3 UCL, London, United Kingdom

340 The use of in-silico research in the development of thoracic and abdominal soft tissue surgical devices: A systematic review

Sanghera J. 1
Pattani N. 2
Mills E. 3
Bolton W. 4
Harji D. 5
Burke J. 6
1 Walsall Manor Hospital, Walsall, United Kingdom
2 St George’s University Hospitals NHS Foundation Trust, London, United Kingdom
3 Mid and South Essex NHS Trust, Essex, United Kingdom
4 School of Medicine, University of Leeds and Leeds Hospitals, Leeds, United Kingdom
5 Department of Colorectal Surgery, Manchester University NHS Foundation Trust, Manchester, United Kingdom
6 Leeds Institute Medical Research, University of Leeds, Leeds, United Kingdom

346 Safety and efficacy of genicular artery embolization for knee joint osteoarthritis associated pain: A systematic review

Othman R.
Fawzy N.
Alfaisal University, Riyadh, Saudi Arabia

436 The effects of timing, surgeon expertise, and advanced laparoscopic techniques on patient outcomes in laparoscopic emergency surgeries for acute cholecystitis

Hreish Y.
Gregory G.
Nottingham University Hospital Trust, Nottingham, United Kingdom

446 Use of eye tracking to understand the trainee–trainer relationship: a systematic review

Kennett J.
UHS, Southampton, United Kingdom

460 Acute chest syndrome in post-operative sickle cell disease patients: A systematic review of predisposing factors and interventions

Khalaf Z. 1
Mahmood M. 2 3
1 Royal Blackburn Hospital, Blackburn, United Kingdom
2 Trinity Centre for Health Science, Dublin, Ireland
3 Tallaght University Hospital, Dublin, Ireland

